# Possible Adverse Effects of High-Dose Nicotinamide: Mechanisms and Safety Assessment

**DOI:** 10.3390/biom10050687

**Published:** 2020-04-29

**Authors:** Eun Seong Hwang, Seon Beom Song

**Affiliations:** Department of Life Science, University of Seoul, Dongdaemun-gu, Seoulsiripdae-ro 163, Seoul 02504, Korea

**Keywords:** nicotinamide, methylnicotinamide, NAD^+^, adverse effect, PARP, SIRT1, SIRT3, mitochondria, reactive oxygen species, DNA methylation

## Abstract

Nicotinamide (NAM) at doses far above those recommended for vitamins is suggested to be effective against a wide spectrum of diseases and conditions, including neurological dysfunctions, depression and other psychological disorders, and inflammatory diseases. Recent increases in public awareness on possible pro-longevity effects of nicotinamide adenine dinucleotide (NAD^+^) precursors have caused further growth of NAM consumption not only for clinical treatments, but also as a dietary supplement, raising concerns on the safety of its long-term use. However, possible adverse effects and their mechanisms are poorly understood. High-level NAM administration can exert negative effects through multiple routes. For example, NAM by itself inhibits poly(ADP-ribose) polymerases (PARPs), which protect genome integrity. Elevation of the NAD^+^ pool alters cellular energy metabolism. Meanwhile, high-level NAM alters cellular methyl metabolism and affects methylation of DNA and proteins, leading to changes in cellular transcriptome and proteome. Also, methyl metabolites of NAM, namely methylnicotinamide, are predicted to play roles in certain diseases and conditions. In this review, a collective literature search was performed to provide a comprehensive list of possible adverse effects of NAM and to provide understanding of their underlying mechanisms and assessment of the raised safety concerns. Our review assures safety in current usage level of NAM, but also finds potential risks for epigenetic alterations associated with chronic use of NAM at high doses. It also suggests directions of the future studies to ensure safer application of NAM.

## 1. Introduction 

Since Elvehjem et al. reported a cure for canine pellagra dogs using nicotinic acid amide (known as nicotinamide) in 1938 [1], nicotinamide (NAM) at doses far above those required as a vitamin (e.g., 500–3000 mg) has been applied to various diseases and conditions. Its usefulness has been observed in protection of neurons and vascular cells from ischemic reperfusion and similar oxidative stresses [2], prevention of neurological dysfunctions such as Alzheimer’s and Parkinson’s diseases [3,4,5,6,7,8,9], and treatment of depression and other psychological disorders [10,11]. The compound also showed promise against development of uremic pruritus, inflammatory diseases [12,13,14], photo-aging and skin cancers [15], and even HIV reproduction [16,17] (summarized in Table 1). The effectiveness of NAM on preventing onset of diabetes mellitus has been shown in some human studies [18,19] although in later studies absence of such effect were also reported [20,21]. Some early studies also showed improvement of psychological conditions such as depression and anxiety in humans upon NAM treatment [22,23]. NAM is currently used against skin diseases such as bullous pemphigoid [24], skin cancers [25], and acne lesions [26]. Furthermore, it is widely added as an effective ingredient in various cosmetic products [27]. (Examples of human study results are shown in Table 2). 

Since the early 1940s, NAM and niacin (also known as nicotinic acid (NA)) have been heavily consumed as food supplements in the USA. Per capita consumption of niacin in the USA has doubled since implementation of mandatory niacin fortification in the early 1940s [51]. Due to the growing demand for NAM in food additives, cosmetic supplements, and dietary pills, its production has continuously grown. Furthermore, a recent increase in public awareness on the beneficial effects of NAM has caused substantial growth of its production, from 56.520 metric tons in 2012 to 71.192 metric tons in 2016, an increase of 30 % (Global Nicotinamide Market Research Report 2017). 

NAM’s efficacy as a therapeutic agent may not be as acute or as strong as that of drugs targeting a particular enzyme. The effects of NAM are, in most part, mediated by increase in the level of nicotinamide adenine dinucleotide (NAD^+^), which plays important roles in metabolic homeostasis, and activities of certain enzymes that play critical roles in health and longevity. SIRT1, for example, is a molecule that plays important roles against age-associated dysfunction as well as in resistance to oxidative stress. Levels of both NAD^+^ and SIRT1 activity decline with aging. Counter-balancing this change through NAD^+^ supplementation has been proposed as beneficial for healthy aging in animal and human studies [52]. Therefore, long-term intake of NAM as a dietary supplement is tempting. This trend demands an assurance of safety and a clear understanding of the mechanisms of action of NAM. Literature reviews have presented various beneficial effects of NAM and the underlying mechanisms (e.g., Song, 2019) [27]. However, there is a current lack of assessment on NAM’s potential adverse effects. Studies reporting such effects are scattered, and a comprehensive analysis has not yet been performed. This review presents a collective literature search on the possible adverse effects of NAM and offers a comprehensive analysis on the possible underlying molecular mechanisms.

## 2. Briefs on Biochemistry Associated with Mechanisms Underlying NAM’s Positive Effects

**Figure 1:**
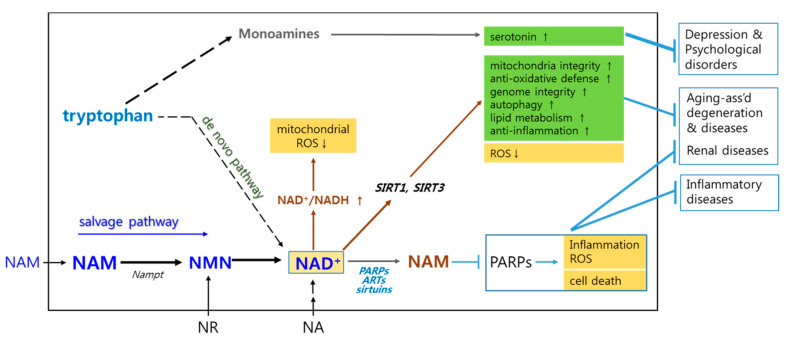
A schematic representation of routes of metabolism and beneficiary effects of NAM. In cells (grey-lined box), NAM is converted to nicotinamide adenine dinucleotide (NAD^+^) mainly through salvage pathways via nicotinamide mononucleotide (NMN). NAD^+^ is reduced to become NADH and thereby, establishes NAD^+^ redox, through which NAM treatment affects mitochondrial energetics and ROS generation. NAD^+^ is also broken down to NAM and ADP-ribose by poly(ADP-ribose) polymerases (PARPs), sirtuins, and a family of ADP-ribose transferases (ARTs), to which NAM exerts feedback inhibition. Among these, inhibition of PARPs constitutes an important route of anti-inflammatory, anti-oxidative, and pro-cell survival effects. Increased level of NAD^+^ activates sirtuin proteins such as SIRT1 and SIRT3, which exert a variety of cell-beneficiary effects such as anti-oxidation, genome stability, autophagy, and lipid metabolism. In addition, they together maintain mitochondria quality and integrity, and thereby, keep reactive oxygen species (ROS) generation at low level. Through these, NAM may exert effects against aging-associated degeneration and diseases, and renal and inflammatory diseases. Through these effects, NAM may help protecting neurons and pancreatic β-cells. Meanwhile, a minor portion of cellular NAD^+^ pool is provided through de novo synthesis from tryptophan, which is also a source for serotonin. Therefore, NAM supplement helps maintaining serotonin level, and thereby alleviates depression and psychological disorders. NAD^+^ level is also elevated through supplementation of nicotinamide riboside (NR) and nicotinic acid (NA).

In human cells, NAM is readily converted to nicotinamide mononucleotide (NMN) and then to nicotinamide adenine dinucleotide (NAD^+^). This salvage pathway for cellular NAD^+^ production appears to be a key route in many of the NAM effects. In support of this, most of NAM’s effects are abolished by inhibition of nicotinamide adenine mononucleotide phosphotransferase (Nampt) [53], which catalyzes NAM conversion to NMN (Figure 1). In addition, the effects of other NAD^+^ precursors, namely NA and nicotinamide riboside (NR), overlap largely with those of NAM.

Cellular NAD^+^ level and, thereby, the NAD^+^/NADH ratio affect not only ATP synthesis, but also generation of reactive oxygen species (ROS) [54,55]. Therefore, NAD^+^ is believed to be involved in aging as well as many pathological conditions [56]. NAM-mediated increase in NAD^+^ level lead to reduced ROS generation from mitochondria through multiple mechanisms (summarized in Figure 1). First, elevation in NAD^+^/NADH leads to reduced mitochondrial membrane potential, decreasing the chance for superoxide generation through retro-transport of electrons [55,57]. Second, high NAD^+^ level facilitates activation of proteins involved in mitochondria quality control, such as SIRT1 and SIRT3, NAD^+^-dependent deacetylases functioning in metabolic homeostasis, cell survival and proliferation, stress resistance, and mitochondria maintenance [27,58]. SIRT1 activity facilitates mitochondrial maintenance by promoting their turnover through mobilization of factors involved in mitochondria biogenesis (peroxisome proliferator-activated receptor gamma coactivator-1α (PGC-1)α) and mitophagy (autophagy gene proteins, ATGs) [27]. It also facilitates cellular anti-oxidative defense through activation of forkhead box protein O (FoxO) proteins, which induce manganese-dependent superoxide dismutase (MnSOD) and catalase [59]. Meanwhile, SIRT3 activity suppresses mitochondrial ROS generation through activation of MnSOD [60,61]. It also promotes mitochondrial integrity by inducing closure of the mitochondrial permeability transition pore (mPTP) through deacetylation of mitochondrial matrix protein cyclophilin D [62]. These anti-oxidative and pro-mitochondrial effects of SIRT1 and SIRT3 proteins function critically in protection of neurons and other cells that are otherwise vulnerable to oxidative stress [2,63,64] and in extension of in vitro replicative life spans of human cells, including bone marrow stem cells [55,65,66,67,68]. Third, NAM’s anti-oxidative effects may also be driven by direct inhibition of poly (ADP-ribose) polymerases (PARPs). PARPs protect genome integrity by binding to DNA at the sites of strand breaks and recruiting DNA repair enzymes. In doing so, they degrade NAD^+^ and generate NAM and ADP-ribose [69,70,71,72]. Activated PARPs induce inflammation through activation of nuclear factor-κB (NF-κB) [73]. By acting as a feedback inhibitor to PARPs, NAM attenuates inflammation and thereby exerts anti-oxidative and therapeutic effects against inflammation-associated diseases [38,74,75,76]. NAM also protects cells with severe DNA damage from apoptotic or necrotic death caused by PARP-induced depletion of NAD^+^ and ATP pools. Indeed, attenuated death of pancreatic β-cells in animals upon NAM administration is proposed to be driven by PARP inhibition-mediated cell protection, enhanced mitochondrial integrity, and reduced ROS generation [77,78]. 

Activation of SIRT1 brings in many other therapeutically useful effects by deacetylation-mediated activation of target molecules. Examples include: enhanced lipid degradation and fatty acid oxidation through activation of peroxisome proliferator-activated receptor (PPAR)-α and -γ [79,80,81]; protection of genome integrity through activation of Ku70, a member of the DNA repair machinery [82]; and suppression of inflammation and apoptosis through inhibition of NF-κB [83] and p53 [84]. Together, these effects can protect against age-associated degeneration and diseases [85]. Inhibition of PARPs and NF-κB by NAM as well as NAD^+^-mediated SIRT1 activation alleviate inflammation, oxidative stress, fibrosis, and apoptosis of renal cells, which are key etiological problems of kidney disease [86]. 

NAM administration-induced elevation of NAD^+^ level supports renal and fecal Pi excretion and alleviates hyperphosphatemia, which is frequently associated with end-phase renal insufficiency [87]. NAD^+^ inhibits Na+-phosphate cotransporters (NaPi2a and NaPi2b), major routes for phosphate uptake in renal proximal tubule and intestine [88,89].

Finally, NAM has been shown to ameliorate depression and psychological disorders. It was also shown effective against a wider spectrum of mental disorders and other complaints including migraines, dizziness, motion sickness, bipolar and behavioral disorders, alcohol dependence, and trophic deprivation-mediated neuronal death [90]. NAM may act in such circumstance by elevating levels of monoamine neurotransmitters including serotonin, a mood-stabilizing hormone that is synthesized from tryptophan. A minor portion of cellular NAD^+^ is supplied by de novo synthesis from tryptophan [91]. NAM supplementation would lower the need for de novo NAD^+^ synthesis and thereby reserve tryptophan for continued serotonin synthesis [92]. 

## 3. Potential Toxicity and Adverse Effects of High Doses of NAM

**Figure 2:**
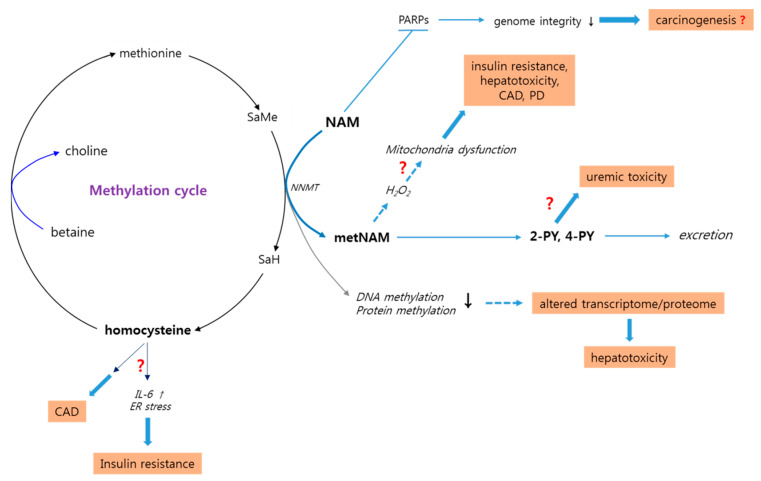
Routes of possible adverse effects of high doses of NAM. High-level NAM has been proposed to induce disorders through multiple different routes. First, NAM inhibits PARP proteins, thereby inducing anti-inflammatory effects but also causing genome instability, which may lead to carcinogenesis. Second, methylation of large amounts of NAM can lower the cellular methyl pool. This leads to reduced methylation of DNA and proteins, thereby changing patterns of gene expression and protein activity. This is proposed to be etiologically linked to liver steatosis and fibrosis. Meanwhile, NAM methylation yields metNAM, which, by inducing high level ROS generation and subsequent dysfunction of mitochondria, may cause development of insulin resistance, coronary artery disease (CAD), and Parkinson’s disease (PD). However, it is not clear whether metNAM-induced ROS generation is harmful. MetNAM is further metabolized to N-methyl-2-pyridone-5-carboxamide (2-PY), whose elevated blood levels can be toxic to patients with chronic renal problems. Meanwhile, increase in the methylation cycle by high-level NAM would lead to an increase in the level of homocysteine, which, by upregulating interleukin-6 (IL-6) expression and inducing endoplasmic reticulum (ER) stress, may trigger insulin resistance and CAD. However, most of these proposed links between high-level NAM and the conditions are poorly supported by experimental evidence.

NAM at doses near 5 mM exerts positive effects on viability and replication potential of cells in culture [65,93]. However, at doses above 20 mM, NAM causes apoptotic death, with a reported half inhibitory concentration (IC50), or half-cell-killing dose, of 21.5 mM [94,95]. Therefore, in vitro, there is a narrow gap between the effective and toxic doses of NAM. Animal studies have shown the lethal dose in 50% of the sample (LD_50_) of NAM to be 2.5 g/kg for oral administration and 2.05 g/kg intraperitoneal administration in mice and slightly higher in rats [96]. These doses approach 150 and 120 g, respectively, when extrapolated to humans. The lethality of these high doses of NAM can be attributed to osmotic shock from the hypertonic solution rather than to biochemical effects. Meanwhile, tolerance of doses near 1 g (or up to 3 g) of daily intake of NAM, even during long-term administration, has been demonstrated in many studies. Examples are the European Nicotinamide Diabetes Intervention Trial (ENDIT), where children were treated with 25–50 mg/kg a day for 5 years without reported adverse effects [20,97] and a recent clinical study which showed that intake of 1.5 g NAM twice daily for 6 months was safe in elderlies [NCT00580931]. These studies assure safety in the current wide-spread practice of long-term dietary intake of 500–1000 mg per day. Although headache, dizziness, and vomiting occurred in healthy humans when NAM at doses up to 6 g was ingested on an empty stomach [43], these adverse effects were minor and resolved upon termination. However, NAM administration above certain levels has shown adverse effects in animals. Moreover, the biochemical nature of NAM raises concerns on potential adverse effects of administration. For this reason, both NAM and its metabolites need to be analyzed in depth for their properties and mechanisms of action (summarized in Table 3 and Figure 2).

### 3.1. Possible Genotoxicity and Carcinogenicity: Inconclusive Effects of NAM 

NAM inhibits the activity of PARPs, which function in recognizing and inducing repair of DNA strand breaks. Therefore, suppression of PARP activity raises reasonable concerns on loss of DNA integrity or enhanced sister chromatid exchange (SCE) [98], which may causatively be associated with certain types of cancers [99]. For this reason, the carcinogenic and co-carcinogenic effects of NAM have been the focus of early studies. However, results in large are not conclusive in both the cases. For instance, treatment with 1–10 mM NAM or other PARP inhibitors, but not with NA, resulted in an increase in SCE [100,101], and 25 mM NAM treatment induced a large structural aberration of chromosomes in Chinese Hamster Ovary cells [102]. On the other hand, NAM deficiency augmented the SCE-inducing potential of PARP inhibitors [103]. This may suggest a PARP-independent effect of NAM in chromatid protection. SIRT1 activation-mediated protection of DNA integrity [82] could be involved which was not considered at the time of the study. Meanwhile, when the mutagenic potential of NAM was assessed using Ames tests with Salmonella or Chinese Hamster fibroblasts, the results showed a null effect [104]. In addition, an increase in DNA repair was reported after gamma- or UV-radiation upon treatment with NAM at doses near 3 mM [105]. Studies on co-carcinogenic effects of NAM are also inconclusive.

For example, pancreatic islet tumors were formed in 64 % of rats intraperitoneally administered NAM (350 mg/kg) and streptozotocin together, while one or no tumors was formed in rats given either NAM or streptozotocin alone [107]. However, a significant reduction of renal adenoma by co-treatment with NAM was reported by the same group [115]. Meanwhile, NAM (350 mg/kg, i.p), when treated with the carcinogen diethylnitrosamine increased the incidence of kidney tumors but decreased that of liver tumors [108]. This suggests an organ-specific co-carcinogenic effect of NAM, but the underlying reasons are unknown. NAM may act differently at different stages of tumor initiation and tumor promotion. Overall, the effects of NAM on carcinogenesis or tumor promotion are not clear, raising considerable concerns regarding long-term intake of NAM. Perhaps, a follow-up study on the subjects of the ENDIT study, which was carried out more than 20 years ago for 5 years and which touted the safety of high doses of NAM, may provide critical information in this regard. Meanwhile, NAM is being clinically tested for possible anti-carcinogenic effects against lung cancer [NCT02416739].

### 3.2. Inhibition of Sirtuin Activity: An Effect that May Not Be Important In Vivo

Like PARPs, sirtuin family proteins are susceptible to feedback inhibition by NAM. Hence, NAM has been a favored inhibitor in experiments involving SIRT1 activity in certain biological functions [46]. For instance, in a study on the role of SIRT1 in reducing triglyceride levels in differentiating adipocytes, 10 μM NAM attenuated SIRT1-mediated reduction of triglyceride levels [116]. This inhibition, if it occurs in vivo, would result in a serious decline in body functions. However, in many of the studies, the observed inhibitory effects of NAM were not conclusive. For instance, treatment with NAM decreased activities of sirtuin proteins in mouse oocytes when examined at 6 or 12 h after treatment, but this effect was not reproduced when examined at 24 h [106]. Observations indicate that a cellular state of high [NAM] is brief in treated subjects. Upon treatment through peritonea or gavage route, tissue NAM level increased immediately and reached nearly four-fold induction within 10 min (in livers of mice administered by gavage). However, this level then decreased and was sustained at a level nearly two-fold higher than basal [46,117]. Upon dietary intake of high doses of NAM, elevated level in blood is also maintained for 2 hours [46]. Similar to NAM, the level of NAD^+^ was shown to increases from similar early time points and remains at the elevated level for more than 12 h in animal tissues [117] and cultured human cells [65]. Therefore, in cells and animals treated with NAM, activities of sirtuin proteins would briefly be suppressed, soon followed by prolonged activation. This pattern of SIRT1 inhibition by NAM in vivo is expected to be different from that of PARPs. While PARPs are acutely activated upon DNA damage and consume NAD^+^ [118], sirtuins remain minimally inactive and are activated only by high level of NAD^+^ [46]. Hence, when PARP is activated, the presence of a high level of NAM results in acute and substantial suppression of its activation. In contrast, sirtuin activities would be drop only marginally and transiently and elevated upon subsequent increase in NAD^+^ level. Furthermore, the IC50 of NAM is lower with PARP-1 (31 μM) than with SIRT1 (50–180 μM) [46], indicating that PARP-1 is more sensitively affected by NAM administration than is SIRT1.

### 3.3. High NAD^+^/NADH Ratio: Concerns Regarding Energy Metabolism

In a study on human fibroblasts, NAM treatment lowered cellular ATP level by approximately 10% after 3-day treatment at 5 mM [65]. This is due to an increase of NAD^+^ level and the associated decrease in NADH/NAD^+^ in mitochondria, which leads to reduced electron supply for the electron transport chain and a decrease in oxidative phosphorylation [55]. If this effect occurs in vivo, physiological activities and organ functions that demand a high level of ATP could be affected, which raises concerns against unguided intake of NAM. In an early study, a decreased growth rate was reported in rats receiving 1 or 2 g/kg NAM for 40 days [109,110]. NAM treatment did not alter proliferation rates of cells in culture but did affect cell size [55,65,68]. Flow cytometric analyses of human fibroblasts and bone marrow stem cells showed an approximately 10% reduction in forward scattering (an indication of cell surface) upon NAM treatment (S. Song and E. Hwang, data not shown). Meanwhile, in a later study by Handler and Dann [110], the authors suspected that the decreased growth rate was attributed to synthesis of metNAM.

### 3.4. High-Level NAD^+^: Effect on Protein Translation

NAD^+^ is found to be added to the 5’ ends of mRNAs and noncoding RNAs of both prokaryotes and eukaryotes, including humans [50]. Normally, 5’,5’-triphosphate-linked N7-methylguanosine is added at the first nucleotide of mRNA, forming the m7G cap [119], which protects mRNA from degradation and functions as an acceptor for the translation initiation complex [120]. In contrast, NAD^+^-capped mRNAs are poorly translated and degraded rapidly [50]. Although little is known about the function and regulation of NAD^+^-capping in human cells, this may occur more frequently when NAD^+^ is in high abundance. Perhaps NAD^+^ level may link cellular energetics to protein translation efficiency. If this is the case, a high level of NAD^+^ would have a global impact on cellular protein levels, and its intervention may have a serious impact on bodily homeostasis as well as development of disease such as cancer. More information should be gathered to address this safety concern. 

### 3.5. High-Level NAM Methylation: Potential Effects of Altered Methyl Pool 

NAM ingested roughly at 200 μmol (or 0.3 mg/kg) daily (for healthy adults at normal diet conditions) [121] is metabolized mainly in the liver by cytochrome p450. It is first methylated by nicotinamide-N-methyltransferase (NNMT) to N-methyl-nicotinamide (metNAM), which is further metabolized to N-methyl-2-pyridone-5-carboxamide (2-PY) or N-methyl-4-pyridone-5-carboxamide (4-PY). Approximately 17.5 μmol is excreted in urine in the form of these metabolites [122,123]. NAM is also oxidized to nicotinamide-N-oxide or hydroxylated to 6-hydroxy-nicotinamide, but the roles of these metabolites are insignificant in humans or unknown yet. 

NAM methylation affects cellular availability of methyl groups, which are used predominantly to methylate DNA and proteins. DNA methylation at cytosines of CpG sites modulates gene expression [124]. Aberrant DNA methylation is involved in obesity and type 2 diabetes [125,126]. Protein methylation at arginine and lysine residues modulates protein function [127]. For example, histone methylation affects histone’s regulatory roles in gene expression. Adequate levels of the cellular methyl pool and methyl metabolism are therefore important for cellular homeostasis, especially in regulating gene expression. A methyl group is transferred from methionine to S-adenosylmethionine (SAMe) in the S-adenosylmethionine cycle (or methylation cycle). SAMe, in turn, yields the methyl group to DNA and proteins and becomes homocysteine, which is converted to methionine by accepting a methyl group from 5-methyltetrahydrofolate (5-MTHF) in a pathway dependent on betaine or folate [128] (Figure 2). NAM act in this cycle as another acceptor of methyl groups. Nicotinamide N-methyltransferase (NNMT) mediates the transfer of methyl groups from SAMe to NAM (Figure 2). Under normal conditions, the NAM concentration in human blood is 69 μM [129], producing inefficient NAM methylation since the K_M_ of the human NNMT for NAM, at approximately 430 μM, is rather high [130]. However, an increase in NAM level leads to a proportional increase of NAM methylation, which is accompanied by a decrease in plasma betaine concentration [111,131]. In rodents, administration of high doses of NAM caused liver steatosis and kidney hypertrophy, effects that were attributed to depletion of methyl donors [111]. Furthermore, dietary intake of 1 or 4 g/kg of NAM altered the expression of genes involved in methyl metabolism in rats [94]. In these studies, however, the amount of NAM was relatively high, equivalent to 50–200 g in humans. Meanwhile, NAM methylation may be counter-balanced by conversion of NAM to NMN, a reaction mediated by Nampt, which has a low K_M_ for NAM (5 nM [132]). Therefore, at the initial stages of increased NAM in cells, NMN conversion would be dominant over NAM methylation, although this might be an overstatement considering that the K_M_ of Nampt increases nearly 20-fold in the presence of NAD^+^ [133]. Overall, further studies are warranted to analyze the effects of NAM on cellular expression of genes and activities of proteins.

### 3.6. High-Level NAM Methylation: Potential Adverse Effects of Altered Methyl Pool

High levels of NNMT expression and metNAM concentration are reported to be involved in certain diseases and conditions such as obesity, type-2 diabetes, hepatotoxicity, Parkinson’s disease, and cancers (summarized in Figure 2). Therefore, NAM administration raises concerns as an etiologic factor for these pathological conditions. However, it is not clear whether common mechanisms underlie the different conditions.

#### 3.6.1. Insulin Resistance and Metabolic Syndrome

NAM treatment caused a significant decrease in insulin sensitivity in human subjects (2 g/day for 2 weeks) [45] and in animals (1 or 4 g/kg for 8 weeks) [94,134]. Direct involvement of metNAM in insulin resistance was suggested. Administration of either NAM or metNAM to healthy rats led to an increase in the levels of glucose and insulin [134]. In addition, inhibition of NNMT activity, and thereby reduction of plasma metNAM level, reduced body weight and improved inulin sensitivity of obese mice fed a high-fat diet [135]. The mechanism by which metNAM induces insulin resistance is poorly understood. An effect of oxidative stress is considered. High-level metNAM increased plasma level of H2O2 [134], and metNAM, but not NAM, triggered oxidative stress in C. elegans [136]. Meanwhile, NNMT, in production of metNAM, also produces S-adenosylhomocysteine, which is a precursor of homocysteine [137] (Figure 2). Positive associations have been reported between increased plasma level of homocysteine and insulin resistance as well as cardiovascular disease [138,139,140,141]. Elevated level of homocysteine leads to insulin resistance by increasing production of interleukin-6 (IL-6) [142], a suppressor of insulin signaling [143] and by inducing endoplasmic reticulum stress [144], a trigger of insulin resistance [145]. Therefore, metNAM itself or a hyper-methyl metabolism process driven by high-level NAM may be the cause of increased insulin resistance. 

#### 3.6.2. Parkinson’s Disease

Brain cells demand high levels of bioenergetics and are heavily dependent on the salvage pathway for NAD^+^ supply [5]. For this reason, NAM administration is considered to be protective for neuron viability and brain function [27]. For Parkinson’s disease (PD) (and other brain diseases), beneficial effects have been observed. NAM treatment protected dopaminergic neurons and sustained striatal dopamine level in a mouse model of PD [3]. Further, the treatment improved locomotor activity and attenuated dopamine depletion in a mouse model [146]. These outcomes are proposed to be driven by NAM-mediated increase in the level of NADH, a cofactor necessary for dopamine biosynthesis and a reducing power for glutathione that is generally insufficient in PD [147]. Additionally, increased activities of SIRT1 and SIRT3 would exert important positive effects for mitochondrial integrity and function, thereby stabilizing energy homeostasis. However, NAM metabolism has also been linked to neurotoxicity and PD. NAM treatment at 500 mg/kg increased the rate of motor decline and induced development of behavioral deficits in PD rats [114]. Importantly, the levels of both NNMT and metNAM are elevated in the brain of PD patients [148,149]. MetNAM was suggested to induce ROS production by inhibiting mitochondrial complex I, which is subsequently destroyed by ROS [150,151]. Hence, mitochondrial dysfunction by metNAM has been proposed as an etiology of PD. However, a recent study found that metNAM does not affect complex I activity, although it does produce ROS [136]. In that study, metNAM functioned positively in extending nematode life span. Therefore, neither the effect nor the mechanism of PD deterioration by NAM is clear and requires closer analyses. On the other hand, preconception of NAM as an inhibitor to sirtuin proteins might contribute to the lack of understanding. A recent study on SIRT1 activation in a mouse model of Huntington’s disease reported that resveratrol attenuated a decline in mitochondrial function and improved motor coordination and learning. NAM, which was adopted in that study as an inhibitor of SIRT1, was reported to block mitochondria-related transcription and worsen motor phenotypes [9]. However, data on such negative effects of NAM were inconsistent with assays that showed NAM treatment to has effects similar to those of resveratrol. In addition, the dose of NAM may not be high enough to produce effects as positive as those of resveratrol. 

#### 3.6.3. Cardiac Diseases 

Serum levels of metNAM and NNMT activity are associated with severity of coronary artery disease (CAD) in human patients [152]. Poor mitochondrial function has been causatively linked to heart failure and CAD; therefore, an adverse effect on mitochondrial function could be linked to CAD [153]. metNAM-mediated increases in ROS and mitochondrial dysfunction could be possible mechanisms [150], although this seems improbable or incomplete, as described for PD above. On the other hand, a high level of homocysteine is a risk factor for CAD [154]; therefore, methyl metabolism, rather than metNAM per se, could be linked to CAD. Meanwhile, nicotinamide riboside (NR), another NAD^+^ precursor, has been shown to effectively protect the heart while preserving mitochondrial ultrastructure and reducing ROS and cardiomyocyte death [155,156], suggesting a cardio-protective effect of high-level NAD^+^. The finding that NR treatment increases the level of metNAM (by more than 10-fold compared to NAM-induced increase in marginal level) [156] could rule out an adverse effect of metNAM per se.

#### 3.6.4. Liver Toxicity 

High doses of NAM showed hepatotoxicity in humans and rats [47,112]. The liver is the organ that expresses NNMT at the highest level [157] and may therefore be more sensitive to high concentrations of NAM. ROS-independent mechanisms have been proposed for hepatotoxicity. A dose-dependent decrease in the level of DNA methylation was observed in the liver cells of rats given dietary supplementation of NAM at a dose of 1 g/kg [94], suggesting interference in cellular methyl metabolism. Meanwhile, NAM fed alongside a high-fat diet caused fatty liver deterioration in mice overexpressing NNMT [113]. NNMT over-activation lowered hepatic NAD^+^ content and decreased SIRT3 activity, and thereby, inhibiting the expression of genes related to fatty acid oxidation. However, this is not expected to occur in cases of NAM treatment. The same study also showed increased expression of the connective tissue growth factor (CTGF) gene through a decrease in methylation of the promoter, and this might be related to liver steatosis and fibrosis. Meanwhile, NAM administration was well tolerated in more recent studies, which generally used purer preparations of NAM [121]. This suggests that hepatic abnormality rather than toxicity might originate from NA [158] that was present as a contaminant, but this possibility has not been carefully addressed. Meanwhile, there are studies reporting NAM’s protective effect against hepatotoxicity caused by alcohol [159]. Ethanol increases the cellular NADH/NAD^+^ ratio, and NAM may counterbalance this by increasing NAD^+^ level.

### 3.7. Potential Positive Effects of metNAM: Contradiction to the Proposed Adverse Effects 

In men experiencing an energy deficit, metNAM level in circulation is increased through elevated NNMT expression and stimulated lipolysis [160]. This suggests that enhanced fat utilization in low-energy conditions is facilitated by metNAM. In cultures of human myotubes, metNAM was secreted and stimulated lipolysis without affecting the level of glucagon or insulin secretion [160]. Therefore, metNAM is proposed to function as a myokine that enhances utilization of energy stores. This is a contradiction to the observation of metNAM-induced obesity or metabolic syndrome [152,161]. In addition, both NAM and metNAM expanded the life span of C. elegans even in the absence of sir-2.1, a worm homologue of SIRT1 [136]. metNAM is a substrate of aldehyde oxidase in cytochrome P450 in generation of H_2_O_2_, which may act as mitohormetic ROS that promote longevity in worms. In this case, metNAM is viewed to produce ROS at levels suitable for mitochondrial function. Furthermore, a direct effect of metNAM on SIRT1 has been suggested. Supplementation of a high-fat diet with metNAM decreases serum and liver cholesterol and liver triglyceride levels in mice. In addition, MetNAM stabilizes the SIRT1 protein and enhances deacetylation of SIRT1 targets. Protection of SIRT1 from proteasome-mediated degradation by metNAM has been suggested [162]. Decreased synthesis of triglycerides and cholesterol in hepatocytes, and thereby amelioration of fatty liver, by high-level metNAM were also reported [163]. 

Meanwhile, intravenous treatment of rats with metNAM at 3–100 mg/kg, but not of NAM, exerted a sustained thrombolytic effect mediated by elevated synthesis of prostacyclin, a vasodilator, by cyclooxygenase-2 activation [164]. 

### 3.8. N-Methyl-2-Pyridone-5-Carboxamide: A Potential Uremic Toxin 

MetNAM is degraded to 2-PY and 4-PY, which are excreted through urine [48]. 2-PY is a major species in urine after intake of NAM or NA [165]. However, it is retained in renal failure patients and thereby classified as a uremic toxin [166]. The toxicity has been presumed to be attributed to PARP inhibition [167], but there is lack of evidence. 2-PY has drawn attention since its level was found to be elevated upon NAM administration to treat hyperphosphatemia, which is a chronic, toxic retention of inorganic phosphorus (Pi) in the blood of chronic kidney disease (CKD) patients [49,87]. NAM treatment alleviates hyperphosphatemia by increasing renal and fecal Pi excretion [88,89]. Meanwhile, 2-PY does not appear to be toxic in individuals without renal dysfunction. In a study of a large group of skin cancer patients treated with NAM at 500 mg twice daily for 5 years, no cases of renal dysfunction or uremic toxicity were reported [168]. 

## 4. Concluding Remarks and Perspectives

Despite numerous studies on the beneficial effects of NAM, not many clinical applications have been made. A reason for this is likely the marginal efficacy of NAM as a therapeutic agent or medicine as compared to drugs targeting specific enzymes. It is rather expected to be more effective against age-associated conditions considering the decline of NAD^+^ levels, sirtuin activities, and mitochondrial quality with aging. In addition, attenuation of ROS generation by NAM can be beneficial in preventing ageing-associated degenerative diseases. These advocate NAM’s usefulness as a long term supplementation. For this reason, any adverse effects of NAM should be disclosed and their molecular mechanisms should be understood. As summarized in Table 2, adverse effects reported for humans are limited to several organs, namely liver, kidney, and cells in plasma. Pancreatic β-cells might also be affected. However, as described, concerns based on cell biological studies for the potential adverse effects of NAM are not limited to those caused by direct inhibition of PARPs (and other ADP-ribosyltransferases). Subsequent changes in NAD^+^ redox as well as the levels of NAD^+^ and NAM metabolites are possible causes of concern, especially for cases of long-term use of NAM. 

This review suggests some points for the direction of prospective studies on the clinical and supplementary usage of NAM. First, effect of NAM on diabetes has been controversial. Both an improvement in β-cell function and a reduction in insulin sensitivity were reported in human studies (Table 2). NAM is still an attractive candidate in the regimen against diabetes, and so conclusive researches need to be made on the effectiveness of NAM on diabetes. Second, although most of the reported adverse effects of NAM resolved upon termination of use [169], extended or life-long use poses a potential threat of irreversible conditions such as carcinogenesis, PD, and type 2 diabetes. In this regard, follow-up with subjects of previous long-term studies such as ENDIT would provide valuable information. Third, there may be issues that cannot be resolved by such retrospective studies. Although life-long NAM supplementation did not produce cancer, it promoted carcinogen-induced tumorigenesis in rats. Furthermore, NAM supplementation to pregnant rats caused changes in the patterns of DNA methylation and mRNA expression in fetal organs [170]. These studies warrant careful examination of the genetic and epigenetic effects of NAM treatment. Fourth, more human studies are demanded. Most of these reported adverse effects occur only in doses higher than those used clinically or for daily dietary supplementation. The reported effects on animals occur with doses at least 10-fold higher than the highest used in human subjects, a 3 g/day dietary intake. Therefore, these adverse conditions and outcomes are not expected to be induced by general pharmaceutical or therapeutic doses of NAM. Still, there exist differences in model animals and humans not only in life span and life style but also in drug metabolism. For example, rodents and humans have different circadian rhythms, which affect NAM-NAD^+^ metabolism differently [171]. These certainly make it difficult to predict the effects of NAM through simple extrapolation of animal studies. Another factor that calls for more studies on human subjects is individual- and age-related variations in pharmacogenetics of NAM metabolism. For example, the enzymatic activity of NNMT in humans varies over five-fold likely due to epigenetic polymorphism [172]. Therefore, understanding on pharmacogenetics and pharmacoepigenetics of the enzymes involved in NAM-NAD^+^ metabolism should accompany the studies on NAM’s efficacies.

Finally, a number of clinical trials on NAD^+^ precursors, i.e., NAM riboside (NR) and nicotinamide mononucleotide (NMN), have been completed or are ongoing [ABOUTNAD (https://www.aboutnad.com/human-clinical-trials)]. Comprehensive understanding of the effects of these chemicals and NAM would produce invaluable information regarding a life-long application of these NAD^+^ precursors.

## Figures and Tables

**Table 1:** Summary of beneficial effects of high doses of nicotinamide (NAM).

Effects	Examples of Effects	References
	Protection against ATP depletion	[2]
	Decreased AD pathology and cognitive decline	[10]
	Improved sensory and motor neurological behavior	[3]
	Increased recovery from bilateral frontal brain injury	[4]
Neuroprotection	Prevention/delay of ischemic stroke in stroke-prone hypertensive rats	[5]
	Reduced lateral geniculate nucleus neuronal death	[6]
	Attenuated hippocampal neuronal death after global ischemia	[7]
	Improved motor deficits associated with Huntington’s disease phenotype	[8]
	Increased NAD^+^ level and mitochondrial function	[9]
Amelioration of depression and psychological disorders	Amelioration of depression	[28]
	Increased social interaction	[11]
Anti-inflammation	Attenuated neutrophil recruitment in carrageenan-induced pleurisy or in lesions of autoimmune disease	[12,13]
	Reduced arthritis activity	[14]
Protection against vision and hearing loss	Attenuated retinal pigment cell death and age-related macular degeneration in animals	[29]
	Reduced incidence of optic nerve degeneration and glaucoma	[30,31]
Immune modulation	Improved mouse survival after lethal Staphylococcus enterotoxin B challenge	[32]
Skin protection/anti- skin disorders/cosmetic effects	Downregulation of the expression of inflammatory cytokines and protection against UV light	[33]
Anti-fibrosis	Attenuated development of pulmonary fibrosis	[34,35]
Anti-metastasis and adjuvant cancer therapy	Decreased growth and progression of bladder tumors	[36,37]
	Photo-protection and reduced incidence of skin cancers	[15]
Anti-HIV and -AIDS	Decreased provirus integration	[16]
	Decreased viral RNA expression	[17]

**Table 2:** Positive and negative effects of NAM shown in human studies ^1^.

Affected Organs and Conditions ^2^	Observed Effects	Dose and Duration	References
**Beneficial effects**			
Joints	Reduced itching in uremic patients	550 mg twice a day (4 weeks)	[38]
pancreatic β-cell	β-cell function preserved and improved	25 mg/kg daily intake (4 weeks)	[18,39]
	Reduced the rate of diabetes incidence	500 mg twice per day (2.5 years)	[19]
	No effect on the incidence of being diabetes-free	1200 mg daily intake (5 years)	[20]
	Ineffective in prevention or delaying clinical onset of diabetes	1.2 g daily intake (3 years)	[21]
Skin	Reduced acne lesions and severity	4% gel applied twice daily (8 weeks)	[26]
	Attenuated immunosuppression with alterations in metabolism and apoptosis	5% lotion applied before UV exposure	[40]
Psychology	Improvements against depression	0.5–1.5 g daily intake (3 weeks)	[22]
	Relief from anxiety	A dose of 2 ug 3 h prior to test	[23]
Kidney	Lowered serum concentrations of phosphorus, parathyroid hormone, and LDL, and increased serum HDL	500 mg/day (with and increment every 2 weeks) (12 weeks)	[41]
Skin cancers non-melanoma	Reduced incidence of various types of skin cancers and actinic keratoses	500 mg twice daily (4 months)	[42]
**Adverse Effects**			
Minor effects	Frontal dull headaches, nausea, headache, dizziness	1–18 g immediate	[43,44]
Pancreatic β-cell/plasma	Decreased insulin sensitivity, increased oxidative stress (H_2_O_2_)	2 g daily (2 weeks)	[45,46]
Liver	Parenchymal-cell injury, portal fibrosis and cholestasis, liver injury	3, 9 g daily (10 days)	[47]
Lymphocytes, platelets	Uremic toxicity-related cancer and thrombocytopenia	1300, 1500 mg daily (24 weeks)	[48]
Kidney/platelets	Decreased serum phosphorus and thrombocytopenia	0.52–2 g daily (3–6 months)	[49,50]

^1^ Some examples of human studies are presented. For more information on the beneficiary effects, check Reference [27]. ^2^ In all human applications except for skin, NAM was administered through dietary intake.

**Table 3:** Examples of adverse effects of high dose NAM reported in studies of cells and animals.

Subjects	Examples of Effects	Dose	Duration	Ref.
	Death of mouse embryonic stem cells	20 mM	3–4 days	[95]
	Tumorigenicity. DNA damage, and sister chromatid exchanges	1–10 mM 10 mM	3 h 40 h	[100,101]
Cells		25 mM	48 h	[102]
	Decreased SIRT1 activity. Increased intracellular ROS, spindle defects, and mitochondria dysfunction	5 mM	6, 12, 24 h	[106]
	Blocked mitochondria-related transcription. Worsened motor disturbance in Huntington’s disease model	0.5, 1 mM	96 h	[9]
Mice and Rats	Oxidative DNA damage in hepatic and renal tissues. Impaired glucose tolerance and insulin sensitivity	1 or 4 g/kg, d.w.	8 weeks	[94]
	Increased lethality	4.5 g/kg, d.w., 2.5 g/kg, i.p.	40 days	[44]
	Occurrence of pancreatic islet cell tumor	350 mg/kg, i.p.	226 days	[107]
	Increased incidence of kidney tumors	350 mg/kg, i.p.	until die	[108]
	Decreased growth rate	1, 2 %, d.w. 1, 2 %, d.w.	24 days 20 days	[109,110]
	Growth inhibition, methyl deficiency, reduced tissue choline level, and increased hepatic lipids	6, 20, 60 mg/100 g bw, i.p.	2, 5 weeks	[111]
	Amelioration of acetaminophen-induced biochemical changes but occurrence of hepatotoxicity in healthy animals	500 mg/kg, i.p.	1.5 h	[112]
	Development of hepatic steatosis and fibrosis	1%, d.w.	6 weeks, 7 months	[113]
	Neurodegeneration of dopaminergic neurons Behavioral deficits and structural brain changes	500 mg/kg, i.p.	28 days	[114]
	Blocked mitochondrial-related transcription, worsened motor phenotype	250mg/kg/day, s.c.	28 days	[9]

i.p., intraperitoneal injection; d.w., drinking water; and s.c., subcutaneous injection.
